# Lessons learned from the “Goodie Box”: A message design study developed and evaluated in community settings for cervical cancer prevention

**DOI:** 10.3389/fonc.2022.935704

**Published:** 2022-09-08

**Authors:** Soroya Julian McFarlane, Susan E. Morgan, Nick Carcioppolo

**Affiliations:** ^1^ University of Georgia, Athens, GA, United States; ^2^ University of Miami, Coral Gables, FL, United States

**Keywords:** cervical cancer, screening, message design, self-test, low resource setting

## Abstract

Despite the availability of free pap testing services, Jamaican women have low human papillomavirus (HPV) screening rates; 16% of women in the Kingston Metropolitan Area have been screened within the prior 3 years. This paper discusses the testing of theory-based messages to increase HPV screening uptake in a low-resource setting, using HPV self-test kits designed for this intervention. A total of 163 Jamaican women, aged 30–65 years, who had not had a pap test in at least 3 years, from two low socioeconomic status communities in Kingston, were enrolled and assigned to one of two versions of an HPV self-test kit, either with or without culturally targeted fear appeal messages. The uptake of screening was high across conditions; 95.6% of participants used the HPV self-test and returned their kits. However, surprising variations were observed in self-test acceptability, explained by differing attitudes toward the message conditions. Based on the results, we recommend four key components to increase HPV screening in low-resource settings: 1) focus on perceived threat in message design, 2) avoid written materials due to literacy concerns, 3) use culturally appropriate interpersonal or community-based channels, and 4) consider alternative solutions (such as a self-test) available at no or low cost to address structural barriers.

## The Goodie Box as follows: Lessons learned from a message design quasi experiment to increase cervical cancer screening in Kingston, Jamaica

The particularly high incidence and mortality of cervical cancer in Jamaica has been documented over decades of research (1–6), and cervical cancer continues to be a national public health priority. Jamaican women have been found to have higher prevalence of human papillomavirus (HPV), the virus that leads to cervical cancer, than found in earlier studies in Jamaica, in close-by English-speaking islands, and in certain countries in other regions (3). Cervical cancer mortality rates are also high—15.8 per 100,000 or approximately 185 deaths per year, representing almost half of those diagnosed annually (7). Ninety percent of these cervical cancer deaths in Jamaica happen because the women had never been screened (1). Despite this, routine screening is not practiced by most Jamaican women (8–11). Over one-third of Jamaican women have never had a pap smear test procedure (11). Only about 22% of Jamaican women have had a pap smear test within the prior year (12), with some parishes reflecting a screening coverage within the past year as low as 15% (11, 12).

Low screening has generally been associated with structural barriers, such as socioeconomic status, lack of access to screening, low education, and lack of knowledge in various contexts (see 13–19). However, the results of various studies in Jamaica hint at other factors at play. Free pap testing is generally available at nearby community clinics and public health facilities (11, 20), and although having insurance is correlated with increased screening, no differences in uptake of services was found across all socioeconomic groups (21). Additionally, while more formal education is associated with increased screening uptake, educated women still have low screening rates; less than 25% had been screened within the past year (21). Jamaican women do have low knowledge and awareness (9, 11). In one study, exposure to an educational session was associated with increased screening intentions from 82% to 96.2% (20). However, since screening intentions were already high at pretest, this begs the question of why actual screening behavior rates remain so low.

Addressing this question may warrant looking beyond individual and health system factors, to understanding the cultural conceptualizations of health and illness that impact screening behaviors. For example, fear of pain was found to be a significant concern for Jamaican women (20). Almost half the women (47%) who had never had a pap smear test report feelings of fear of the test, including fear of pain, compared to less than a third of women (31.7%) who had experienced a pap test (11). Fear of the pap test results was also noted as a barrier to uptake for Jamaican women with experience of the test as well as those without, although there was still a statistically significant higher fear among the latter group. (1, p. 9) explains that, “most women have heard of the pap smear but believe its purpose is to detect rather than prevent cervical cancer”. Further, there is also a misconception among Jamaican women that the pap test itself causes cervical cancer (as cited in 22).

Additionally, low perceived susceptibility to cervical cancer and perceived severity of the disease is associated with a decreased likelihood to screen in Jamaica (11), where cultural beliefs mean that individuals only perceive an illness to be present if there are symptoms (as cited in 23). In a study on Jamaican women aged 15 to 49 years, 56.9% had never even had a pelvic examination, some offering the explanation that they were “healthy and have no sign of gynecological problems” (9, p. 480). There is also low confidence to screen, as women find the pap test embarrassing, even after an educational intervention (20; as cited in 22).

## The extended parallel process model

These findings above cumulatively suggest that a focus on messages that increase perceived susceptibility and perceived severity, including knowledge and awareness of the disease, and increase confidence to screen as an effective prevention measure, might encourage screening uptake. According to the Extended Parallel Processing Model (EPPM), an individual at risk can be influenced to adopt preventative strategies through exposure to messages that increase their perceived threat and perceived efficacy. Perceived threat includes susceptibility (personal risk) and severity (detrimental outcome) of the disease. Perceived efficacy includes self-efficacy (confidence in personal capacity) and response efficacy (confidence in treatment or intervention) to effect desired change (24, 25). These message features ideally lead the individual to ‘danger control’—they feel fear, then process and accept the prescription for preventing the illness that is detailed in the messages. If the threat and efficacy messages are not balanced, it can lead individuals to ‘fear control’—they process the message and reject it and as such do not conform to the recommended behavior change. Additionally, individual traits such as personality can lead an individual to perceive neither threat nor efficacy in the message, and therefore there is no impact that would lead to behavior change (24, 25). The development of the EPPM was informed by the fear as acquired drive model (26, 27); (27), the parallel process model (28), and protection motivation theory (29, 30).

Despite some debate about the effectiveness of this approach to message design, a meta-analysis by (31) showed that all the message characteristic manipulations (fear, severity, susceptibility, self-efficacy, and response efficacy) changed behavior positively based on the strength of the appeal. The results of another meta-analysis revealed that not only were fear appeals effective but also they did not backfire and cause unintended consequences, based on the studies in the review (32).

## Cultural targeting

Although some studies have attempted to understand the EPPM’s capacity to explain culture-specific reactions to fear and threat in minority populations in the United States, Asian, and African contexts (32–37), questions remain. One such question is how cultural differences may explain cognitive processing of threat and the related behavioral outcomes. In the current study, we consider how the implications of cultural manifestations of threat may have important potential in explaining variances in screening behaviors within a population.

“Cultural sensitivity is the extent to which ethnic/cultural characteristics, experiences, norms, values, behavioral patterns and beliefs of a target population as well as relevant historical environmental and social forces are incorporated in the design, delivery and evaluation of targeted health promotion materials and programs” (38, p. 11). A culturally targeted message design therefore attempts to account for cultural nuances in the approach to the development of material targeting a particular cultural context. Cultural targeting triggers cognitive mechanisms (such as attraction and comprehension) by appealing to an individual’s preexisting communication preferences. This not only makes successful message processing more likely but can impact psychological antecedents of behavior that increases the chance for a positive message impact, including changing attitudes, outcome expectations, and improving self-efficacy (39). Further, research that explicitly tests culturally targeted compared with non-targeted communication material among diverse populations is needed to demonstrate its effectiveness and justify its deployment in health promotion interventions (38, 40, 41). In the modified model EPPM (23), Witte demonstrates including certain “universal” cultural variables; however, to the authors knowledge this model has not been further developed or tested.

## Methods

### The intervention—culturally targeted fear appeal messages

We therefore designed a culturally targeted fear appeal message for the Jamaican context, with a focus on culture-based ‘contextualization’, which involves framing “one’s message in a context that is meaningful to the recipient” (39, p. 459) in order to encourage cognitive processing of the messages. To understand if this message would be effective, we developed two versions of an HPV self-test kit—with and without culturally targeted fear appeal messages—and tested them in a field experiment among Jamaican women aged 30 to 65 who had not had a pap smear test in at least 3 years.

The following hypotheses and research questions, based on the EPPM, were tested:

Hypothesis 1: Exposure to the culturally targeted fear appeal message will produce higher self-test acceptability, than those exposed to the no message (plain kit) condition.
Hypothesis 2: Women in the culturally targeted fear appeal condition will exhibit higher (a) perceived efficacy and (b) perceived threat when compared to those in the no message (plain kit) appeal condition.
Hypothesis 3: Message condition and self-test acceptability will be mediated by (a) perceived efficacy and (b) perceived threat.

Additionally, to understand if there was a relationship between culturally targeted fear appeal messages, attitudes, and behavior, we asked the following research questions:

RQ1: What is the relationship between message condition and kit attitudes?
RQ2: Is the indirect effect of message type on self-test acceptability conditional on kit attitudes?

The current study aimed to determine the efficacy of these culturally targeted fear appeal messages to increase screening uptake in this population using an experimental design in which one group received a self-test kit with no message appeals (control) and another group received a self-test kit with culturally targeted fear appeal messages embedded into the design of the kit (intervention). Before conducting the experimental study, a pilot test of the culturally targeted fear appeal messages was conducted with Jamaican women in focus groups. This step of the research was important to determine if the messages drafted by the researchers were, in fact, perceived as intended (manipulation check). Using the Extended Parallel Process Model (EPPM; 24, 25), we developed cervical cancer fear appeal messages that included threat (disease susceptibility and disease severity) and efficacy (self and response efficacy), at surface and deep levels of culture as outlined by Resnicow etal. (38). We integrated feedback on these initial messages from scientific experts on cervical cancer, a community partner organization, and focus groups with Jamaican women into the final messages to be used on a self-testing kit. This process of theory informed message design and the result of this evaluation is beyond the scope of the current manuscript and is described in much further detail elsewhere (see 42).

The culturally targeted fear appeal self-test kit was graphically designed to appeal to Jamaican women and featured illustrations, vibrant Jamaican colors, and a diagram explaining cancer progression. The control group received a plain white self-sampler kit with no message appeal; the only text was the words “Cervical Pre-Cancer/Cancer (bold); Self-sampler Screening Test (regular). The self-test kits in the two conditions included (1) a cotton swab, methanol-based solution, biobag, and hand sanitizer and (2) instructions for using and returning the kit. The instructions were also culturally targeted in the intervention condition, while the control condition received standard, non-targeted instructions (see 42 to view the designs). Beyond these differences, the experimental conditions were designed to be as similar as possible in terms of packaging and placement of text.^1^

### Site of experiment and participants

This study took place in two communities in Kingston, Jamaica, a developing country in the English-speaking Caribbean. No significant sociodemographic differences were found between the communities in income, education, marital status, and religiosity, although the control group was younger with higher employment rates (see **Table 1** for comparison of communities). Across both communities, participants were aged 30 to 65; the mean age of participants was 42.87 (SD = 9.895). About 65.6% participants were employed, 83.3% earned less than $JA30,000 (USD$300), and 77.9% did not have health insurance. The majority of women who participated had at least a high school education (68.8%), and some had a technical diploma or college degree (21.9%); 9.4% had less than a high school education. More than half of the women (55.2%) were single and had never been married; 38.1% stated that they were either married or living with their significant other; and 4.7% of the women were separated or divorced. The majority (83.4%) shared that they considered themselves to be religious or that religion was important to them, and many (69.9%) spent between more than once a week to once a month participating in religious activities. The inclusion criteria targeted women who were not up to date on their routine pap smear examinations; the participating women had not been screened for at least 3 years. More than 50% (N = 83) of the women who participated had their last pap test between 3 and 6 years ago, 20% (N = 34) between 7 and 22 years ago, and 15% (N = 25) had never had a pap test in their lifetime.

**Table 1:** Participant socio-demographic characteristics by intervention condition.

	Control Community N = 89	Intervention Community N = 74	p-value*
		Mean (SD)	
Age, years	41.09 (10.222)	45.01 (9.099)	.01
		n (%)	
Income (monthly)			
Less than JA$15,000	34 (54.8%)	25 (39.1%)	.13
JA$15,001 - $30,000	21 (33.9%)	25 (39.1%)	
More than JA $30,000	7 (11.3%)	14 (21.9%)	
Employment			
Employed/self-employed	51 (58%)	56 (77.8%)	.03
Retired/homemaker	3 (4.2%)	5 (5.7%)	
Unemployed	13 (18.1%)	32 (36.4%)	
Insurance			
Not insured	76 (93.8%)	51 (77.3%)	.00
Insured	5 (6.2%)	15 (22.7%)	
Education			
<High School	8 (9.2%)	7 (9.6%)	.31
High School	56 (64.4%)	54 (74.0%)	
>High School	23 (26.4%)	12 (16.4%)	
Marital Status			
Single/never married	48 (57.5%)	42 (62.7%)	.44
Living with significant other/married	37 (43.5%)	25 (37.3%)	
Religious?			
Yes	72 (82.8%)	64 (88.9%)	.27
No	15 (17.2%)	8 (11.1%)	
Religious Importance			
Very or somewhat unimportant/Unsure	14 (13.5%)	10 (15.9%)	.67
Somewhat or very important	74 (84.1%)	64 (86.5%)	
Religious Involvemeny			
More than once a week/once a week/once a month	61 (68.5%)	53 (71.6)	.67
Only special occasions/never	28 (31.5%)	21 (28.4%)	

*Totals may not equal 163 due to missing values. Percentage totals exclude participants who omitted the question.

### Procedure

Purposive and snowball sampling was used to recruit women in order to meet the requirements of the community-based study. The final sample for data analysis consisted of 163 women (89 in the control community; 74 in the intervention community) after eligibility screening and data cleaning in line with the inclusion criteria. (43) suggests that bootstrapping is sufficiently robust to support a sample size of less than 100 per condition for mediation analysis since it facilitates resampling with replacement of data, with correction for bias (44). A ‘toss of the coin’ method was used to randomly assign the standard-of-care, plain self-test kit to the control community and the culturally targeted fear appeal kit to the intervention community. The University of Miami Institutional Review Board and the Jamaican Panel on Ethics and Medico-legal Affairs, Ministry of Health, approved this study. This study was also registered on clinicaltrials.gov.

During data collection, outreach workers were hired to recruit participants from their own communities. They distributed promotional flyers and invited eligible women to enroll and participate in the study through door-to-door visits. The project team explained the goal of the research to potential participants as a study that aimed to understand if Jamaican women would use an HPV self-test to screen for cervical cancer. Data collection took place at a community church, a community center, and a basic school over the course of 2 weeks. Eligible women were encouraged to refer their female friends and family members from the same communities to the project. All participants were screened for eligibility, after which the PI obtained written informed consent.

Participants went through the following steps to complete the study: (1) completion of a baseline survey (demographic, sexual and reproductive background, knowledge and attitudes about HPV/cervical cancer); (2) a brief individual or small group sensitization session on the importance of cervical cancer screening conducted by the PI using a short intervention/educational script; (3) completion of a short survey on social proliferation and screening intentions; (4) using and returning of the self-test kit version they received at home or in the clinic bathroom; (5) completion of a posttest survey upon returning their samples. After completing all the steps, participants received a small incentive of $21USD ($2,500 Jamaican dollars). All returned HPV self-tests were sent to the Laboratory for Clinical and Biological Studies at the Sylvester Comprehensive Cancer Center at the University of Miami. The clinical results from the self-tests are beyond the scope of the current paper (45).

### Measurement

Seven-point Likert-type response scales were used to assess participants’ responses (from strongly disagree to strongly agree), except where noted. Self-test acceptability was the dependent variable. Cognitive and affective variables from the fear appeal theory included perceived threat (susceptibility and severity) and perceived efficacy (self-efficacy and response efficacy). The authors also gathered data on participants’ attitude toward the kit (‘kit attitudes’) as well as control (participant background and demographic) variables. These measures are described briefly below.

### Self-test acceptability

Acceptability of the self-test was measured using an 11-item scale (α = .86, M = 6.07, SD = .94); an example was “I would recommend using the self-test to my female family members and friends”.

### Threat

Susceptibility was measured using three items including, “It is likely that I will develop cervical cancer” (α = .81, M = 4.35, SD = 1.77). Severity was measured with a two-item scale after removing a weaker item. The scale included “I believe that cervical cancer is a severe health problem” (α = .67, M = 5.76, SD = 1.28).

### Efficacy

Self-efficacy was measured and analyzed using three items (α = .68 M = 5.55, SD = 1.24). An example of an item was: “Doing a screening test like pap smear or HPV test is easy for me”. Response efficacy was measured using three items, including “Screening tests like pap smears or HPV tests can save lives by catching cervical cancer early” (α = .83, M = 5.90, SD = 1.19).

### Kit attitudes

This six-item scale was created for the current research and included items such as “The instructions on the kit about how to use the self- test were too complicated”. The reliability was (α = .67, M = 6.08, SD = .79).

### Control variables

Control variables included sociodemographic variables (age, sex, ethnicity/race, education level, household income), prior sexual activity (whether the participant had ever had sexual intercourse), and prior health behaviors (ever had ever had an abnormal pap test, had an HPV infection in the past, had genital warts, or been diagnosed with cervical, oral, or anal cancer). Prior sexual activity and prior health behaviors was measured with yes, no, don’t know, or refuse response options.

### Data analysis

REDCap (Research Electronic Data Capture) (http://project-redcap.org/), a web-based tool for clinical researchers, was used to capture data from the field. Statistical Package for Social Sciences (SPSS) was used for data analysis.

## Results

The uptake of screening was high across conditions; 95.6% of participants used the HPV self-test and returned their kits. Since self-sampler uptake was so high, it was not statistically meaningful to pursue uptake as an outcome variable. Instead, self-sampler acceptability was used in any analyses as an outcome variable.

### Experimental hypotheses 1, 2, and 3

Hypothesis 1 predicted that exposure to the self-test kit with culturally targeted fear appeal messaging would produce self-test acceptability than those exposed to the self-test kit with no message appeal. Results from the ANCOVA indicated that there was no significant difference in self-test acceptability [F(1,147) = 2.97, p = 0.09] between the conditions.

Hypothesis 2 predicted that women in the culturally targeted fear appeal condition would exhibit higher (a) perceived efficacy and (b) perceived threat when compared to those in the no message appeal condition. To assess if there were differences between the groups on self-efficacy, response efficacy, perceived relevance, perceived susceptibility, and perceived severity, a statistical mediation analysis was conducted using SPSS PROCESS Model 4. Results demonstrated that there was no significant difference between conditions in perceived efficacy [F(1,148) = .12, p = .73] and a marginally significant difference between the groups in perceived threat [F(1,148) = 3.65, p = .06]. However, by examining perceived severity of the disease (a construct within perceived threat), there were significant differences between conditions [F(1,148) = 4.88, p = .02]. Comparing the estimated marginal means showed that respondents in the control/no message appeal condition reported higher perceived severity (M = 5.96) than respondents in the culturally targeted fear appeal condition (M = 5.47). As such, the opposite effect of what was hypothesized occurred; perceived severity was higher in the community that received the testing kit that did not have printed messages on the box, than women who received the culturally targeted fear appeal message.

Hypothesis 3 predicted that message condition and self-test acceptability would be mediated by (a) perceived efficacy and (b) perceived threat. Regression analyses were conducted revealing that message condition was not a significant predictor of perceived efficacy, b = -.08, SE = .17, p = .64, and that perceived efficacy was not a significant predictor of self-test acceptability, b = .12, SE = .07, p = 08. Additionally, message condition was not a significant predictor of perceived threat, b = .33, SE = .20, p = .10. Perceived threat was, however, a significant predictor of self-test acceptability b = .12, SE = .05, p = .03. Despite this relationship, the results did not support the overall mediational hypothesis.

### Research questions 1 and 2

RQ1 investigated the relationship between message condition and kit attitudes. A one-way ANCOVA was conducted to compare the impact of the message condition on kit attitudes, controlling for age and employment. Results from the ANCOVA indicated that there was a significant difference between conditions in kit attitudes [F(1,147) = 8.00, p = .01]. Comparing the estimated marginal means showed that the control (no message condition) had more positive kit attitudes (M = 6.24) than the culturally targeted fear appeal condition (M = 5.86). Therefore, culturally targeted fear appeal messages on the kit were not viewed as positively as no message appeal at all.

**Figure 1:**
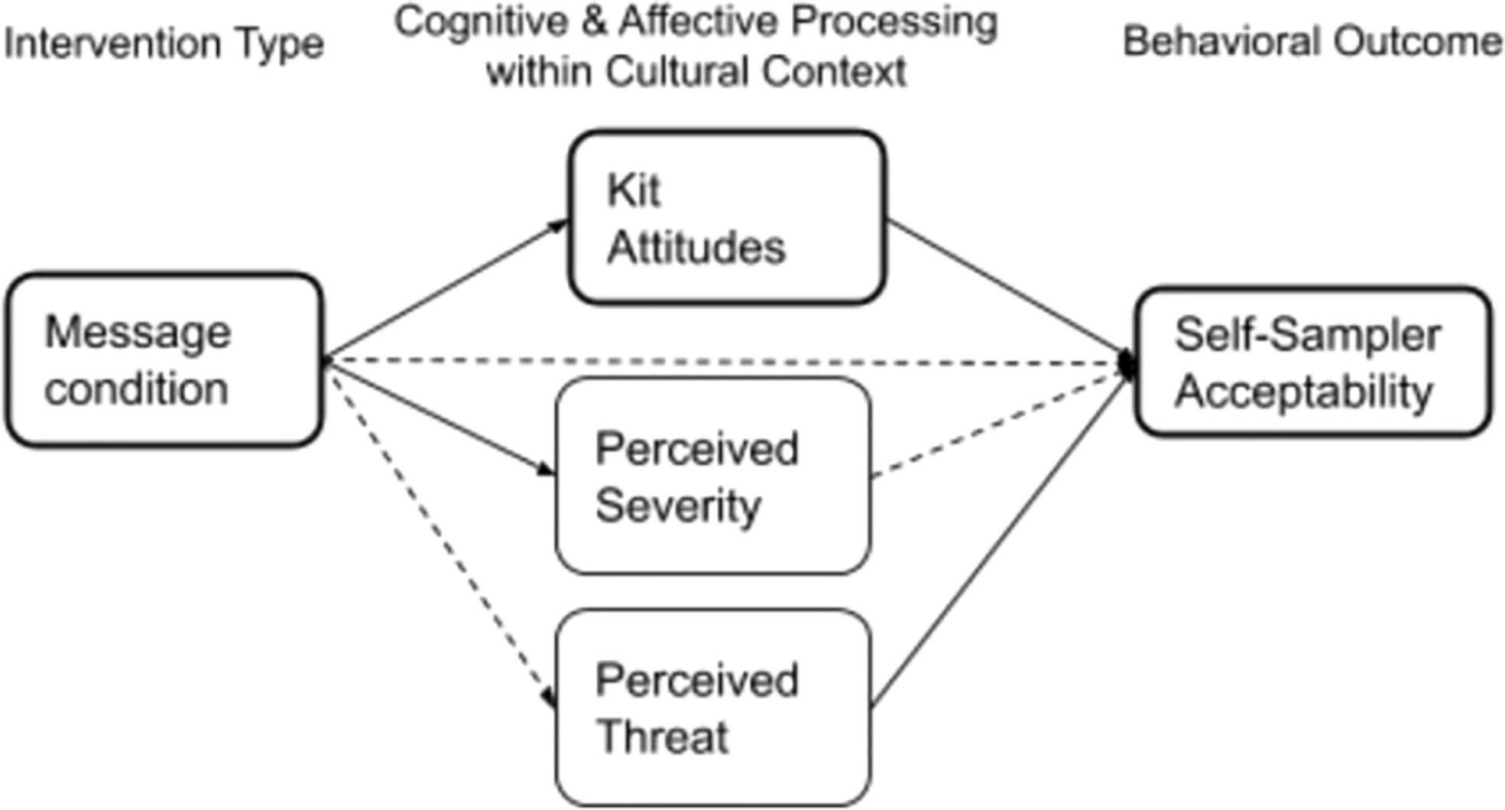
Mediations N-148. Mediation model (PROCESS Model 4) with message condition as the independent variable, and perceived threat, perceived severity and kit attitudes as mediators. Only mediators with one or more significant paths are depicted. Solid lines indicate significant paths (p< .05).

RQ2 investigated the indirect effect of message type on self-test acceptability conditional on kit attitudes. Regression analysis was used to investigate if kit attitudes mediated the effect of message condition on self-test acceptability, controlling for age and employment. Results indicated that message condition was a significant predictor of kit attitudes, b = .38, SE = .34, p = .01, and kit attitudes was a significant predictor of self-test acceptability, b = .81, SE = .07, p <.001. Message condition was no longer a significant predictor of self-test acceptability after controlling for the mediator, kit attitudes b = .28, SE = .17, p = .09, consistent with full mediation. Approximately 46% of the variance in self-test acceptability could be explained by the predictors (R^2^ = .46). The indirect effect was tested using a bootstrap estimation approach with 10,000 samples. These results indicated that the indirect coefficient was significant, SE = .09, 95% CI = .07,.61. Message condition was associated with approximately.3 points higher self-test acceptability scores as mediated by kit attitude (see **Figure 1**).

## Discussion

“Persuasion researchers have recognized for some time that it is easier to demonstrate attitude change in the laboratory than in the field” (46). Despite this, it is still incumbent on applied researchers to continue to utilize theory to understand real-world challenges through field experimentation (47). This research aimed to build on the EPPM to understand how culturally targeted fear appeal message characteristics are mediated by related cognitive processes, which ultimately influence attitudes, beliefs, and behavior. Despite the practical contributions of the study toward educating and screening 177 women who had not had a pap test in 3 to 40 years, or had never had a pap test at all, the current study was not able to explain what specific message features supported the success of the intervention. However, an indirect effect of message condition on self-test acceptability was observed, explained by differing attitudes toward the kit.

One might ask why attitudes toward the kit are important, if ultimately, the screening behavior was overwhelmingly positive. Women who received no message appeal had more positive kit attitudes and increased acceptability of the self-test than those who received the culturally targeted fear appeal message self-test kit. We believe that more positive attitudes toward the control kit and higher acceptability might be explained by literacy challenges among the participants—this, since the kit attitudes survey items measured the extent to which participants thought the self-test kit text was complicated, easy, or took too long to read. It would follow that participants therefore preferred the condition in which they did not have additional reading outside of the self-test instructions. Although not measured formally, there was anecdotal evidence of low functional literacy in this population, since an unusually high number of women claimed to have forgotten their glasses during data collection (a red flag of illiteracy in cancer prevention studies, see 48).

On the other hand, we believe self-test acceptability was still high across both conditions due to interpersonal communication support from the study team in reading the survey items and the kit to individuals, the brief oral education session by the PI, and the overall community-based project approach. Research has demonstrated the influence of message channels as a moderator of cultural targeting on persuasion, in that audio/video has stronger effects than print or mixed media (49). Prior research has also demonstrated the effectiveness of utilizing social networks (50) and social organizations like churches (51, 52) to disseminate messages and increase uptake of intervention, particularly in diverse communities. We therefore suggest that future studies should minimize the use of text (focus on audio/video formats) and adapt a community-based and interpersonal approach for health communication in this cultural context.

An additional significant finding was that women who received the ‘no message appeal’ self-test kit had higher perceived severity than the women who received the severity messages deliberately embedded in the culturally targeted fear appeal kits. Since both groups received a self-test kit, this can only be explained by the difference in presentation of the kit and its messages. We believe that the women may have instead perceived the control kit to have a more clinical appearance, which may have induced more perceived disease threat, compared to the colorful, culturally targeted design accompanied by fear appeal messages. While this result defies our original hypothesis, this evidence suggests that even a threat message that is not explicit can be considered in message design for cervical cancer prevention in this context. Additionally, cultural targeting may actually reduce the effectiveness of threat in fear appeals, and this needs further consideration and empirical testing.

Further, the provision of the self-test kit to all participants may have intrinsically influenced perceptions of threat and efficacy in participants because the immediate availability of the kit greatly reduced nearly all barriers Jamaican women experience when trying to obtain cervical screening (like identifying a provider, allocating financial resources for the test, and making an appointment, as well as fear of pain).

Therefore, multicomponent cancer communication interventions that consider addressing cultural and structural barriers may have the greatest potential to change behaviors in underscreened populations. Based on the results, the authors recommend four key components to increase HPV screening in low-resource settings: focus on perceived threat in message design; avoid written materials due to literacy concerns; use culturally appropriate interpersonal or community-based channels; and consider alternative solutions (such as a self-test) to be made available at no or low cost to address structural barriers.

### Limitations and future research

Noise in the data, often associated with field research, resulted in challenges controlling for all extraneous variables in order to effectively observe effects and explain the underlying mechanisms leading to those effects (47). The PI, a Jamaican woman who was heavily involved and visible in the project in both conditions, as well as outreach workers who were from each community to assist with recruitment of participants, may have enhanced attitudes and assessments of cultural acceptability across both conditions.

Additionally, the informed consent process, as well as the brief educational session about HPV and cervical cancer, which were administered to every woman across conditions, in retrospect could be seen to both contain threat and efficacy messages. For example, in the educational script, response efficacy could be evident in “Cervical cancer is the easiest gynecologic cancer to prevent with regular screening tests and follow up”. With more controls between conditions, these limitations might be minimized, and a greater effect might have been observed. In addition, the study sample size is small; as such, in order to provide stronger support for the hypotheses presented, a larger range of studies is needed. Therefore, an important step for future research to understand the efficacy of culturally target fear appeals will be to test for each of these potential drivers of uptake (such as outreach workers, educational script, print message) compared with a true control condition (such as a government- or NGO-issued brochure), in a randomized control trial, to further refine a model of culturally targeted fear appeals and to determine the efficacy of specific messages to increase screening uptake.

## Conclusion

The current study has begun the process of examining how cultural and structural barriers can be addressed to positively influence cancer screening behavior. Culturally targeted fear appeal theory-based messages were embedded within an HPV self-test kit and tested in an underscreened, low-income community in a developing country. The results have practical and theoretical implications: first, HPV self-testing has incredible potential to increase efficacy and screening; second, high acceptability of screening may be encouraged by inducing perceived threat and utilizing an interpersonal and verbal (no text) message format to accommodate for literacy challenges. Ultimately, despite the inherent challenges in field research, the widening cancer health disparities affecting vulnerable communities create an imperative for continued work to refine theory-based communication interventions that potentially address the modifiable cognitive, affective, and behavioral factors that influence screening behavior in these contexts.

## Data availability statement

The raw data supporting the conclusions of this article will be made available by the authors, without undue reservation.

## Ethics statement

This study was reviewed and approved by University of Miami Institutional Review Board. The patients/participants provided their written informed consent to participate in this study.

## Author contributions

SJM conceptualized and led the data collection, analysis and write up. SEM contributed to conceptualization, editing and review. NC provided support for methods and data analysis, and review. All authors contributed to the article and approved the submitted version.

## Funding

Funding This research project was funded by The Global Oncology Innovation Grant of the Sylvester Comprehensive Cancer Center at the University of Miami. The authors received no financial compensation for the research, authorship, and/or publication of this article.

## Conflict of interest

The authors declare that the research was conducted in the absence of any commercial or financial relationships that could be construed as a potential conflict of interest.

## Publisher’s note

All claims expressed in this article are solely those of the authors and do not necessarily represent those of their affiliated organizations, or those of the publisher, the editors and the reviewers. Any product that may be evaluated in this article, or claim that may be made by its manufacturer, is not guaranteed or endorsed by the publisher.

## Footnotes

1 “Development Of Content, Format And Messages”, which includes the text for the screening kits and how they were developed, is provided in a supplement.
